# Intraoperative Flow Cytometry for the Evaluation of Meningioma Grade

**DOI:** 10.3390/curroncol30010063

**Published:** 2023-01-07

**Authors:** George A. Alexiou, Georgios S. Markopoulos, Evrysthenis Vartholomatos, Anna C. Goussia, Lefkothea Dova, Savvas Dimitriadis, Stefania Mantziou, Vasiliki Zoi, Anastasios Nasios, Chrissa Sioka, Athanasios P. Kyritsis, Spyridon Voulgaris, George Vartholomatos

**Affiliations:** 1 Neurosurgical Institute, University of Ioannina School of Medicine, 45110 Ioannina, Greece; 2 Department of Neurosurgery, University Hospital of Ioannina, 45500 Ioannina, Greece; 3 Haematology Laboratory—Unit of Molecular Biology and Translational Flow Cytometry, University Hospital of Ioannina, 45500 Ioannina, Greece; 4 Department of Pathology, University Hospital of Ioannina, 45500 Ioannina, Greece; 5 Department of Pathology, German Oncology Center, 4108 Limassol, Cyprus; 6 Department of Nuclear Medicine, University Hospital of Ioannina, 45500 Ioannina, Greece

**Keywords:** meningioma, grade of malignancy, intraoperative flow cytometry

## Abstract

Meningiomas are the most frequent central nervous system tumors in adults. The majority of these tumors are benign. Nevertheless, the intraoperative identification of meningioma grade is important for modifying surgical strategy in order to reduce postoperative complications. Here, we set out to investigate the role of intraoperative flow cytometry for the differentiation of low-grade (grade 1) from high-grade (grade 2–3) meningiomas. The study included 59 patients. Intraoperative flow cytometry analysis was performed using the ‘Ioannina Protocol’ which evaluates the G0/G1 phase, S-phase, mitosis and tumor index (S + mitosis phase fraction) of a tumor sample. The results are available within 5 min of sample receipt. There were 41 grade 1, 15 grade 2 and 3 grade 3 meningiomas. High-grade meningiomas had significantly higher S-phase fraction, mitosis fraction and tumor index compared to low-grade meningiomas. High-grade meningiomas had significantly lower G0/G1 phase fraction compared to low-grade meningiomas. Thirty-eight tumors were diploids and twenty-one were aneuploids. No significant difference was found between ploidy status and meningioma grade. ROC analysis indicated 11.4% of tumor index as the optimal cutoff value thresholding the discrimination between low- and high-grade meningiomas with 90.2% sensitivity and 72.2% specificity. In conclusion, intraoperative flow cytometry permits the detection of high-grade meningiomas within 5 min. Thus, surgeons may modify tumor removal strategy.

## 1. Introduction

Meningiomas are typically benign extra-axial neoplasms that develop from arachnoid meningothelial cells. Meningiomas account for 38.3% of all adult central nervous system tumors, with an incidence of 8.81 instances per 100,000 people [1]. Meningiomas in children are exceedingly rare [2]. According to the WHO’s 2021 classification, meningiomas can be classified as grade 1, 2 or 3 [3]. Grade 3 meningiomas have a high rate of tumor recurrence (60–94%), compared to grade 2 tumors (29–59%) and grade 1 tumors (7–25%). The extent of resection is of prognostic significance [4]. Distinguishing grade 1 from grade 2/3 meningiomas may not be possible based on conventional MRI sequences [5].

Assessment of meningioma grade during surgery is important for surgical strategy in order to reduce postoperative complications by the removal of tumors from neighboring structures such as brain, nerves and vessels. Meningioma grade is typically impossible to determine during intraoperative pathological examination using frozen section analysis. Therefore, it is crucial to develop an intraoperative approach that would allow meningioma grading. Intraoperative flow cytometry (iFC) has been introduced as a method for the assessment of intracranial tumor grade, extent of resection, presence of neoplastic tissue during stereotactic brain tumor biopsy and diagnosis of central nervous system lymphoma [6,7]. Here, we set out to investigate the value of iFC for the assessment of meningioma grade.

## 2. Material and Methods

Patients hospitalized in the Neurosurgical Department of our institution over an 8- year period who were operated on for an intracranial lesion suspicious for meningioma on conventional radiological imaging (MRI/CT scan) and a tumor sample that was available during surgery for intraoperative flow cytometry analysis were included in the study. The researcher who carried out the cell cycle study was unaware of the results from the preoperative CT scan/MRI, the intraoperative findings and the results from frozen section analysis. Diagnosed tumors were graded according to the World Health Organization (WHO) 2007, 2016 classification scheme. Intraoperative flow cytometry analysis was performed using the ‘Ioannina Protocol’ within 5 min from sample receipt as discussed previously in detail [7]. Briefly, a tumor sample from the tumor’s core of 2–5 mm^3^ was used for analysis and the G_0_/G_1_, S and G_2_/Μ phases were quantified, for a cell population of at least 5000 cells. Following DNA analysis, two indices were calculated: tumor index, as the cumulative percentage of cells in S and G_2_/M phases, and DNA index, as the fraction of geometric mean fluorescence in G_0_/G_1_ of tumor cells to that of normal peripheral blood mononuclear cells (PBMCs). Chicken erythrocytes were also used for validation of the cytometer performance. According to flow cytometry analysis, the tumors were categorized as low grade (WHO grade 1) or meningiomas of a higher grade (WHO grade 2/3). Our Institutional Review Board approved the study, and it was in accordance with the principles of the Declaration of Helsinki.

### Statistical Analysis

The G_0_/G_1_, S-phase, mitosis fraction and tumor index (S + G_2_/Μ mitosis fraction) of low-grade vs. high-grade meningiomas were compared using the Mann–Whitney U test. The threshold value effectively distinguishing low-grade from high-grade meningiomas was determined using receiver operating characteristic (ROC) analysis. Area under the ROC curve (AUC) analysis by bootstrap was used for internal validation. Pearson correlation coefficient was calculated between grade, tumor index and ki-67 expression. The mean and standard deviation were used to express continuous data. A probability value of less than 0.05 was used to define the threshold of significance.

## 3. Results

**Figure 1:**
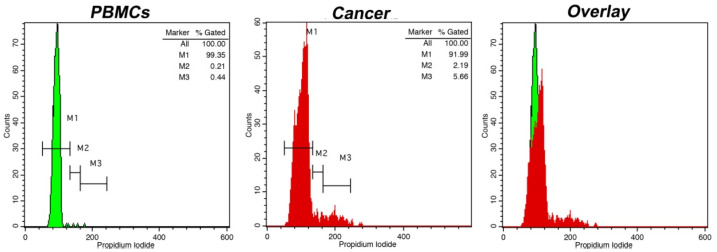
Cell cycle distribution analysis using intraoperative flow cytometry in a grade 1 meningioma. Markers M1, M2 and M3 represent G_0_/G_1_, S and G_2_/M cell cycle phases, respectively (absolute quantification of each marker is presented in the top right corner in each histogram). On the left of each figure the cell cycle distribution of peripheral blood mononuclear cells (PBMCs) is presented as a control. The presented case is diploid, with a DNA index = 1, while the tumor index (i.e., percentage of proliferative cells) was calculated at ~7%. In the overlay histogram, the G_0_/G_1_ peak of cancer cells in red is discernible from that of normal cells in green.

Fifty-nine patients (21 men, 38 women, mean age 57.3 years, range 19–86) met the inclusion criteria for the study. There were 41 grade 1, 15 grade 2 and 3 grade 3 meningiomas. Meningiomas of a higher grade had significantly higher S-phase fraction (median value 4.5 vs. 2, *p* = 0.0021), mitosis fraction (median value 10 vs. 4, *p* = 0.0001) and tumor index (median value 16 vs. 7.3, *p* = 0.0001) compared to low-grade meningiomas (Table 1 and Figure 1). The median expression of Ki-67 is 8% (*n* = 17) in higher-grade meningiomas and 2% (*n* = 29) in low-grade ones (Table 1), based on available immunohistochemistry data, which is significantly different (*p* = 0.001).

**Figure 2:**
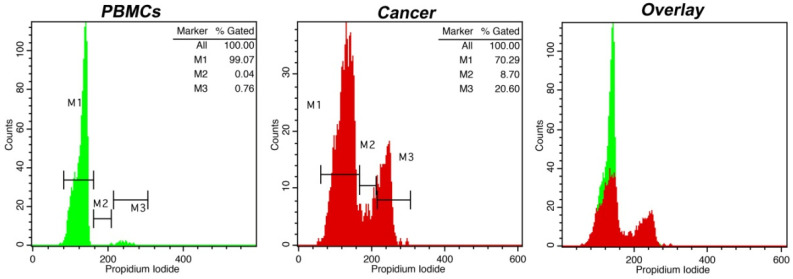
Cell cycle distribution analysis using intraoperative flow cytometry in a higher-grade meningioma. PBMCs and cancer cells are represented as in Figure 1, with respective markers M1, M2 and M3 representing G_0_/G_1_, S and G_2_/M cell cycle phases (presented as quantified in the top right corner of each histogram). The case exhibits a DNA index = 1 and a tumor index = 29%. In the overlay histogram, the G0/G1 peak of cancer cells in red is discernible from that of normal cells in green.

Meningiomas of a higher grade had significantly lower G_0_/G_1_ phase fraction compared to low-grade meningiomas (median value 82 vs. 92.8, *p* < 0.0001) (Figure 2). Thirty-eight tumors were diploids and twenty-one were aneuploids. Nine diploid tumors were grade 2 meningiomas, and the remaining twenty-nine tumors were grade 1. From the 21 aneuploid tumors, 8 were high-grade meningiomas (5 grade 2 and 3 grade 3).

**Figure 3:**
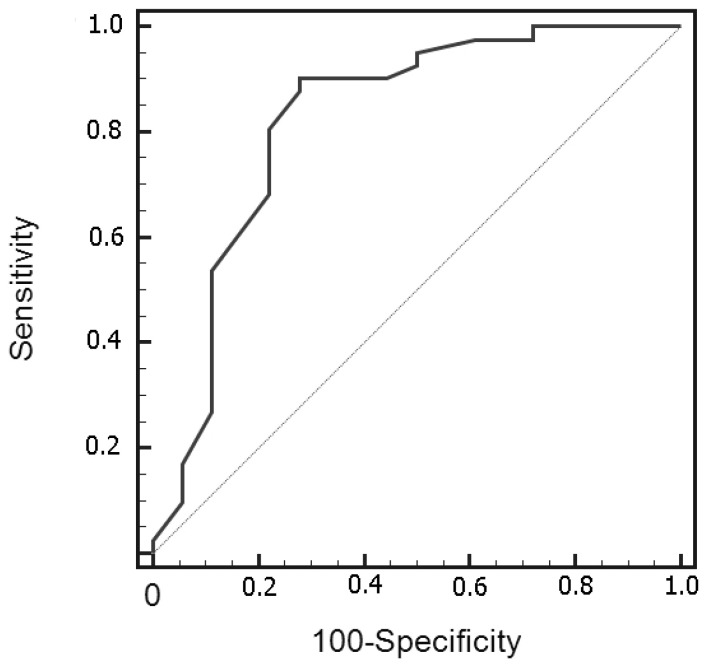
Receiver operating characteristic curve analysis in differentiating grade 1 from grade 2/3 meningiomas, based on tumor index calculation. The area under the curve was 0.825.

No significant difference was found between ploidy status and meningioma grade. ROC analysis indicated 11.4% of tumor index as the optimal cutoff value thresholding the discrimination between low- and high-grade meningiomas with 90.2% sensitivity and 72.2% specificity (Figure 3). All tumors with a tumor index of more than 22% were high-grade. The ROC-curve was validated internally using AUC calculation following bootstrap analysis (1000 replications) and revealed an observed coefficient of 0.7953251 (Table S1).

Significantly, tumor index, the expression of ki-67 and meningioma grade are correlated, based on Pearson coefficient (0.645 between grade and tumor index, 0.512 between grade and ki-67 expression and 0.581 between ki-67 expression and tumor index, *p* < 0.001 in all cases, Table S2).

## 4. Discussion

The present study showed that IFC may permit the detection of high-grade meningiomas intraoperatively with 90.2% sensitivity and 72.2% specificity. All meningiomas with a tumor index of more than 22% were high grade. Although high-grade meningiomas more often were aneuploids, no difference was found between ploidy status and meningioma grade. Significantly, grade, tumor index and ki-67 expression were found to be correlated, providing evidence that iFC is accurate for meningioma grade detection and compatible with Ki-67 analysis.

IFC has recently been introduced as a useful tool for solid tumor evaluation [6,8,9,10,11,12,13]. Cell cycle analysis was one of the first applications of flow cytometry, and when a flow cytometer is available, cell cycle analysis can be performed at virtually no cost. Over recent years, our group developed a protocol (Ioannina protocol) for fast cell cycle analysis that permitted the intraoperative use of this technique [7]. Sample requirements are minimal (2–5 mm^3^), no substance is administered to the patient and the analysis of each sample requires less than 5 min. The resulting histogram provides the percentages of cells in G_0_/G_1_, S and G_2_/M phase fraction. Ploidy status can also be evaluated. Based on that, previous studies have shown that iFC can permit the differentiation of low- from high-grade tumors both in adults and children [7,14,15,16]. The assessment of resection margins for cancerous cells in brain tumor surgery is another important application as well as the verification of neoplastic tissue during stereotactic brain tumor biopsies [7,17]. Via the assessment of cluster of differentiation (CD) markers, central nervous lymphoma can be diagnosed intraoperatively [18]. A real-time intraoperative device based on flow cytometry has also been proposed [19]. IFC has also been proven to be useful during surgery for other solid tumors such as liver cancer, pancreatic cancer, head and neck malignancies, breast cancer surgery, colorectal, gynecological and urological cancers [8,9,10,11,12,13,20].

Before iFC, flow cytometry was performed in meningiomas mainly in paraffin-embedded or frozen tissue using protocols that required a substantial amount of time, usually more than 15–20 min. In a study that included 425 meningioma cases, flow cytometry was performed in paraffin-embedded tissue. An S-phase fraction of more than 10.15% was found to be correlated with decreased recurrence-free survival. Ploidy status was not significantly associated with recurrence-free survival [21]. Lin et al., in a study of 43 patients with meningioma, found that the best cutoff value for the discrimination of grade 1 from grade 2 meningiomas was the G_2_/M-phase and S + G_2_/M-phase fractions of 5.12 and 7.52%, respectively [22]. Sampling was always performed from the tumor’s core. Heterogeneity has been reported previously when sampling was performed from the region close to the dural attachment, the center of the tumor and the peripheral region of the tumor in contact with the brain surface [23]. The correlation of tumor radiological heterogeneity with sampling results is currently under investigation by our group.

The material analyzed was limited, and only in a few cases, we performed repeated measurements to provide a data intra-assay reproducibility, which was high (data not shown) and agrees with the confirmed high reproducibility of the Ioannina protocol used. The limited material may be a limitation of this assay and requires caution from both surgeons and cytometrists in order to provide a proper analysis. Another limitation is the fact that meningiomas have a low proliferation capacity, making it more difficult to differentiate them via iFC than other aggressive tumor types. This may be the main reason behind the fact that the sensitivity and specificity found in this study is the lowest among the available iFC protocols [8,9,10,11,12,13,24].

Using another flow cytometry protocol for intraoperative use that lasts about 9 min, Matsuoka et al. studied 117 meningioma cases. The authors calculated the ratio of the cell number with a higher-than-normal DNA content to the total number of cells. This was named the malignant index (MI). Cell cycle analysis was performed and grade 1 meningiomas could be discriminated from grade 2/3 using a cutoff value of 8% with 64.7% sensitivity and 85.0% specificity [25]. A positive correlation between the Ki-67 index and meningioma annual growth rate, as assessed by serial MRIs, with MI was also found. Oya et al. studied the intratumoral heterogeneity of MI in meningiomas by analyzing samples from the attached, central and peripheral sections of the tumor. The MI in different sample sites was linked to tumor biological characteristics such as annual growth rate and the development of pial feeders [23]. Alexiou et al. sought to correlate meningioma malignancy as assessed by iFC with perfusion, diffusion and diffusion tensor MRI metrics. In a study that included 14 meningiomas (9 grade 1 and 5 grade 2), a significant correlation was found between relative cerebral blood volume (rCBV) and G2/M phase fraction and a negative significant correlation between rCBV and G_0_/G_1_ phase fraction. A significant correlation was observed between fractional anisotropy ratio and G_0_/G_1_ phase fraction. Nevertheless, no significant role of ploidy status was found [24].

Specific molecular alterations have recently been incorporated into the morphology for the diagnosis of WHO grade 3 meningiomas [26]. Interestingly, the presence of TERT promoter mutations has been associated with higher grades and increased recurrence rate [27]. Alterations of CDKN2A and CDKN2B were found more frequently in recurrent meningiomas and were associated with poor prognosis [28,29]. The 2021 WHO classification of CNS tumors incorporates the above molecular events for assessing the histological grading. Therefore, anaplastic meningiomas are now diagnosed if TERT promoter mutations and/or CDKN2A/B homozygous deletion occur, even in the absence of histological features consistent with anaplasia. Future studies will offer novel insights into the molecular mechanisms of meningioma development as well as the role of iFC to predict such molecular alterations.

In conclusion, intraoperative flow cytometry is a novel, operator-independent and low-cost technique that can be implemented during intracranial tumor surgery. IFC may permit the discrimination of low- from high-grade meningioma with high sensitivity and specificity. This information is important for surgeons to modify their strategy if needed. Further studies are needed to verify our results and correlate flow cytometry metrics with recurrence rate.

## Figures and Tables

**Table 1:** Patient characteristics regarding iFC and Ki-67 staining results.

	Grade 1	Grade 2/3
No of patients (%)	41 (69.5%)	18 (30.5%)
Diploid (%)	29 (49.1%)	9 (15.2%)
Aneuploid (%)	13(22%)	8 (13.7%)
G_0_/G_1_ (median)	92.8%	82%
S-phase (median)	2%	4.5%
G_2_/M phase (median)	4%	10%
Tumor index (S + G_2_/M)	7.3%	16%
Ki-67 (median)	2%	8%

## Data Availability

The data presented are available upon request from the corresponding author.

## Supplementary Materials

The following supporting information can be downloaded at: https://www.mdpi.com/article/10.3390/curroncol30010063/s1, Table S1: results of Area Under the ROC Curve (AUC) following Bootstrap (1000 replications) analysis; Table S2: correlation between tumor-index, grade and Ki-67 expression
