# Supplementary material for: Intraoperative Flow Cytometry for the Evaluation of Meningioma Grade
Source: Curr Oncol. 2023 Jan 7;30(1):832–8. doi: 10.3390/curroncol30010063 (PMC9858265; doi:10.3390/curroncol30010063)
Supplement: Supplementary file 1 [file curroncol-30-00063-s001.zip › curroncol-2113853-supplementary.pdf]

Table S1. results of Area Under the ROC Curve (AUC) following Bootstrap (1000 replications) analysis.

**AUC**	Observed Coef.	Bias	Bootstrap Std. Err.	[95% Conf.	Interval]	
	**0.7953251**	**-0.0019977**	**0.0746995**	**0.6489167**	**0.9417334**	(N)
				**0.6354693**	**0.9294736**	(P)
				**0.6330275**	**0.9281986**	(BC)

Table S2. correlation between tumor-index, grade and Ki-67 expression.

Correlations

		**TUMOR-INDEX**	**GRADE**	**Ki-67**
TUMOR-INDEX	Pearson Correlation	1	0.645**	0.581**
	Sig. (2-tailed)		0.000	0.000
	N	59	59	46
GRADE	Pearson Correlation	0.645**	1	0.512**
	Sig. (2-tailed)	0.000		0.000
	N	59	59	46
Ki-67	Pearson Correlation	0.581**	0.512**	1
	Sig. (2-tailed)	0.000	0.000	
	N	46	46	46

****.** Correlation is significant at the 0.01 level (2-tailed).
